# Supplementary material for: Overexpression of Two Members of D7 Salivary Genes Family is Associated with Pyrethroid Resistance in the Malaria Vector Anopheles Funestus s.s. but Not in Anopheles Gambiae in Cameroon
Source: Genes (Basel). 2019 Mar 12;10(3):211. doi: 10.3390/genes10030211 (PMC6472155; doi:10.3390/genes10030211)
Supplement: Supplementary file 1 [file genes-10-00211-s001.pdf]

Table 1. List of primers used in this study.

a- Primers for qPCR analysis for D7 salivary genes from *An. gambiae*.

Gene ID	Genes name	Forward sequence	Reverse sequence	Amplicon size (bp)
AGAP008278	*D7l1*: D7 long form salivary protein 1	5′ TGTGTGTTCCGTGGT TTTCG 3′	5′ TCA GGC TCC AGG TCA CTC TT 3′	101
AGAP008279	*D7l2*: D7 long form salivary protein 2	5′ ACT GAT CGG TCT GCA ACT CT 3′	5′ TCT CCA GCA TTG ATA GAA GCG A 3′	150
AGAP028120	*D7l3*: D7 long form salivary protein 3	5′ TAC GGA GGC AAT GGA AAG GC 3′	5′ TCT GGC ACA CTC TTC AAT GGA 3′	151
AGAP008284	*D7r1*: D7 salivary related protein 1	5′ CGT TAT GAA GGC GCT CGA TT 3′	5′ GTC ATG CTT CCG GTC CTT CT 3′	100
AGAP008282	*D7r2*: D7 salivary related protein 2	5′ AGT ACA CGC CCG TTA GCA G 3′	5′ TTG GCC GCA TGA TTG ACG C 3′	149
AGAP028120	*D7r3*: D7 salivary related protein 3	5′ CGT GCA AGG GAA AGA TAT GGAC3′	5′ CAT TTT CAG CAC CAA GAA GC 3′	112
AGAP008281	*D7r4*: D7 salivary related protein 4	5′ TGA GGA TGG TCG TGG AGA TT 3′	5′ TGT CGG GAC TTG TAC GCA TT 3′	118
AGAP008280	*D7r5*: D7 salivary related protein5	5′ GTG CGG CAG TAT CGT GTA AC 3′	5′ TCC AGT GCT GCT AGA ACG TT 3′	125
AGAP009945	*GADPH*: Glyceraldehyde-3-phosphate deshydrogenase	5′ CTG CAA AAA GTC GAT ACC GC 3′	5′ CCT CGT ACA CGT ACA TCG TGA 3′	102
AGAP010592	*RSP7*: 40S Ribosomal protein S7	5′ AGA ACC AGC AGA CCA CCA TC 3′	5′ GCT GCA AAC TTC GGC TAT TC 3′	98

b- Primers for qPCR analysis for D7 salivary genes from *An. funestus*.

Gene ID	Geme name	Forward sequence	Reverse sequence	Amplicon size (bp)
AFUN007416	*D7L*: D7 long form salivary protein	5′ CGCATGTATATTCCGTGGCTTTC 3′	5′CCTGGAAGCTGCATTTAACGATT3′	109
AFUN016457	*D7r1*: D7 salivary related protein 1	5′ CCAGTAGGTAAACGAGCGAATG 3′	5′CAGCTTCTCTACTGCACCACT 3′	150
AFUN016455	*D7r2*: D7 salivary related protein 2	5′GCGAACACCTTCTATACGTGC 3′	5′ACGGATCACTCAGTCGCATC 3′	112
AFUN016454	*D7r3*: D7 salivary related protein 3	5′ACCTTCTATACGTGCTTCTTGGG 3′	5′ACGGATCACTCAGTCGCATC 3′	106
AFUN016456	*D7r4*: D7 salivary related protein 4	5′AGGAATTGGTGAAGGCTGGA 3′	5′CTTTCTCACTTTCCGACGGC 3′	150
AFUN006819	Act: Actin 5C	5′TTAAACCCAAAAGCCAATCG 3′	5′ TCCGAGTTCATTTCCAGCTC 3′	111
AFUN007153	*RSP7*: 40S Ribosomal protein S7	5′ GTGTTCGGTTCCAAGGTGAT 3′	5′ TCCGAGTTCATTTCCAGCTC 3′	98

c- Primers used for *An. funestus D7r3* and D7r4 DNA amplification and sequencing.

Gene ID	Geme name	Forward sequence	Reverse sequence
AFUN016454	*D7r3*: D7 salivary related protein 3	5′ GGTGTATCCTCTCTTTAGTGA 3′	5′ TTGTACAACAGAAGCAAGGG 3′
AFUN016456	*D7r4*: D7 salivary related protein 4	5′ ACACATATCCCGTAGAGACA 3′	5′ CAGATTAAAGTCGTCGCATG 3′
